# Reduced Influence of apoE on Aβ43 Aggregation and Reduced Vascular Aβ43 Toxicity as Compared with Aβ40 and Aβ42

**DOI:** 10.1007/s12035-020-01873-x

**Published:** 2020-01-17

**Authors:** Lieke Jäkel, Elisanne A.L.M. Biemans, Catharina J.M. Klijn, H. Bea Kuiperij, Marcel M. Verbeek

**Affiliations:** 1 Department of Neurology, Donders Institute for Brain, Cognition and Behaviour, Radboud Alzheimer Centre, Radboud University Medical Center, Nijmegen, The Netherlands; 2 Department of Laboratory Medicine, Radboud University Medical Center, Nijmegen, The Netherlands; 3 Department of Neurology, Radboud University Medical Center, 830 TML, P.O. Box 9101, 6500 HB Nijmegen, The Netherlands

**Keywords:** Amyloid-β (1–43), Apolipoprotein E, Alzheimer’s disease, Cerebral amyloid angiopathy, Aggregation, Cytotoxicity

## Abstract

The amyloid-β 43 (Aβ43) peptide has been shown to be abundantly expressed in Alzheimer’s disease plaques, whereas only relatively low levels have been demonstrated in cerebral amyloid angiopathy (CAA). To better understand this discrepant distribution, we studied various biochemical properties of Aβ43, in comparison with Aβ40 and Aβ42. We assessed the interaction of Aβ43 with the three apoE isoforms (apoE2, apoE3, and apoE4) using SDS-PAGE/Western blotting and ELISA, aggregation propensity using thioflavin T assays, and cytotoxicity towards cerebrovascular cells using MTT assays. We found that Aβ43 did not differ from Aβ42 in its interaction with apoE, whereas Aβ40 had a significantly lower degree of interaction with apoE. At a molar ratio of 1:100 (apoE:Aβ), all apoE isoforms were comparably capable of inhibiting aggregation of Aβ40 and Aβ42, but not Aβ43. All Aβ variants had a concentration-dependent negative effect on metabolic activity of cerebrovascular cells. However, the degree of this effect differed for the three Aβ isoforms (Aβ40 > Aβ42 > Aβ43), with Aβ43 being the least cytotoxic peptide towards cerebrovascular cells. We conclude that Aβ43 has different biochemical characteristics compared with Aβ40 and Aβ42. Aggregation of Aβ43 is not inhibited by apoE, in contrast to the aggregation of Aβ40 and Aβ42. Furthermore, cerebrovascular cells are less sensitive towards Aβ43, compared with Aβ40 and Aβ42. In contrast, Aβ43 neither differed from Aβ42 in its aggregation propensity (in the absence of apoE) nor in its apoE-binding capacity. Altogether, our findings may provide an explanation for the lower levels of Aβ43 accumulation in cerebral vessel walls.

## Background

The amyloid-β (Aβ) peptide plays a central role in Alzheimer’s disease (AD), as it accumulates into senile plaques, one of the neuropathological hallmarks of AD. In 80% of AD patients, Aβ also accumulates in the cerebral vessel walls, a pathology called cerebral amyloid angiopathy (CAA) [1, 2].

The Aβ peptide is thought to be produced through proteolytic cleavage of the amyloid precursor protein (APP) produced by neurons, into peptides of either 48 or 49 amino acids. Further processing, through the sequential release of three amino acids by γ-secretase, leads to two independent production pathways of Aβ40 and Aβ42 (Aβ49 → Aβ46 → Aβ43 → Aβ40 and Aβ48 → Aβ45 → Aβ42) [3–6]. Aβ40 and Aβ42 are the most abundantly produced Aβ isoforms and are produced at a ratio of 9:1 [7]. The Aβ40 species is a frequent constituent of CAA, whereas the more aggregation-prone Aβ42 is the core component of senile plaques in AD [1, 8]. More recently, studies have highlighted the potential of yet another Aβ species, Aβ43, in the pathogenesis of AD. Aβ43, which has an additional threonine at the C-terminus relative to Aβ42, has been shown to be highly abundant in the AD brain and to possess neurotoxic properties [9–11]. However, only relatively low Aβ43 levels have been demonstrated in vascular deposits in CAA [11, 12].

Cerebral vessels consist of endothelial cells, covered by a basement membrane, and vascular smooth muscle cells (SMCs) in the arterioles and arteries or human brain pericytes (HBPs) in the capillaries. Both SMCs and HBPs contribute to brain function, including regulation of cerebral blood flow and blood-brain barrier (BBB) maintenance [13, 14]. In CAA, vascular Aβ initially deposits in the basement membrane and in later stages compromises SMC and HBP viability. Cerebrovascular cells are known to be susceptible to Aβ-mediated toxicity [15–17]. In final stages of CAA, the smooth muscle cell layer in larger vessels is completely replaced by Aβ, the endothelial cell layer is well preserved [18–20].

Apolipoprotein E (apoE) is a protein involved in the regulation of Aβ clearance at the BBB, although the exact mechanisms remain somewhat unclear [21, 22]. apoE exists in three isoforms (apoE2, apoE3, and apoE4) that have different structural conformations as a result of substitution of 1 or 2 amino acids [23]. The possession of an APOE ε4 allele increases the risk of developing CAA or AD [24–26]. The apoE protein might affect Aβ clearance, either by binding to Aβ [27], and thereby affecting its aggregation and clearance, or by binding to receptors that are responsible for Aβ clearance across the BBB, such as the low-density lipoprotein receptor–related protein 1 (LRP1) [28, 29].

We hypothesized that the relative absence of Aβ43 in CAA may be explained by Aβ peptide–specific characteristics. A peptide-dependent interaction between Aβ isoforms and apoE might affect clearance of a specific Aβ peptide at the BBB. Furthermore, levels of Aβ peptides in CAA may be determined by differences in aggregation properties of the peptides that may either promote or prevent peptide accumulation in the vasculature. Finally, a variable vulnerability of cerebrovascular cells towards various Aβ isoforms may also determine the degree to which different Aβ isoforms accumulate in cerebral vessel walls. In this study, we characterized the interaction of Aβ43 with apoE isoforms and the aggregation properties of Aβ43 as well as its cerebrovascular toxicity in comparison with that of Aβ40 and Aβ42.

## Methods

### Preparation of Aβ Peptide and apoE Solutions

Synthetic human Aβ40, Aβ42, and Aβ43 were purchased from Anaspec (Fremont, CA, USA) and monomeric solutions were prepared according to Ryan et al. [30]. In short, Aβ peptides were dissolved in 10% NH_4_OH at 0.5 mg/ml. After 10-min incubation at room temperature (RT), samples were sonicated for 5 min and dispensed into Eppendorf tubes (50 or 100 μg per tube). Samples were snap-frozen in liquid nitrogen and lyophilized to remove the NH_4_OH. Aliquots were stored at − 80°. Immediately prior to use, the peptides were dissolved in 60 mM NaOH followed by immediate dilution in distilled water to a concentration of 288 μM Aβ in 13 mM NaOH. Recombinant apoE, produced in *Escherichia coli*, was obtained from Fitzgerald Industries (Acton, MA, USA) and dissolved in sterile PBS to a concentration of 1 mg/ml. Bradford reagent (B6916, Sigma-Aldrich, St. Louis, MO, USA) was used to determine exact protein concentrations. For this purpose, 2.5 and 5 μl of both Aβ and apoE stock solutions were added to a 96-well plate. Then, 250 μl of Bradford reagent was added and the plate was mixed on a shaker for 30 s. After 15-min incubation at RT, absorbance of the samples was measured at 620 nm, and the absorbance was used to calculate correction factors to ensure equal input of different protein isoforms for further experiments. As we observed major differences (up to 400%) in supplied quantities of commercially obtained peptides that should contain the same amount of Aβ, we found it critical to assess and control for these differences. For the MTT assay, Aβ solutions in 13 mM NaOH were neutralized in 10X Dulbecco’s PBS, yielding a pH of 7.4, before further dilution in culture medium to the desired concentration. For analysis of complex formation with apoE, the various Aβ isoforms (Aβ40, Aβ42, or Aβ43; 50 μM) and the various apoE isoforms (apoE2, apoE3, or apoE4; 500 nM) were co-incubated overnight in PBS at 37 °C.

### ELISA for Aβ-apoE Complexes

A 96-well plate was coated overnight with goat anti-apoE (K74190G, Meridian Life Sciences, Memphis, TN, diluted 1:3000 in PBS) at 4 °C, followed by washing with PBS containing 0.05% Tween20 (PBST) and 2-h blocking with Odyssey blocking buffer (LI-COR Biosciences, Bad Homburg vor der Höhe*,* Germany) diluted 1:1 in PBS. After washing, wells were incubated with the Aβ-apoE protein samples (added in duplicate) diluted 200 times in sample diluent (INNOTEST ß-Amyloid (1-42) ELISA kit; Fujirebio, Ghent, Belgium) for 2 h at RT, while shaking at 600 RPM. Wells were then washed and incubated for 1 h at RT with biotinylated anti-Aβ antibody (mouse-α-Aβ clone 4G8, Biolegend, San Diego, CA; cat. 800701, diluted 1:2500 in PBS containing 1% BSA), while shaking at 600 RPM. Subsequent washing was followed by 30-min incubation with streptavidin-HRP (ThermoFisher, Waltham, MA, diluted 1:60000 in PBST), at RT, with shaking at 600 RPM. After the final washing step, TMB solution (Sigma-Aldrich) was added as a substrate. The reaction was stopped with 1 M H_2_SO_4_. Optical density (OD) values were measured at 450 nm on a Tecan Infinity F50 plate reader.

### SDS-PAGE and Western Blotting for Detection of Aβ-apoE Complexes

SDS-stable complex formation was analyzed under non-reducing conditions. Samples were diluted in concentrated non-reducing sample buffer (62.5 mM Tris-HCl, pH 6.8, 22% glycerol, 2% SDS and bromophenol blue) and separated by electrophoresis on a 12% polyacrylamide gel containing SDS. Proteins were transferred to PVDF membranes by Western blotting. Membranes were blocked using Odyssey blocking buffer (LI-COR), diluted 1:1 in PBS. Staining of the proteins was performed successively for apoE and Aβ, by incubation with goat anti-apoE (1:2500, overnight at 4 °C, Meridian Life Sciences, Memphis, TN) followed by donkey anti-goat Alexa-680 (1:5000, 1 h at RT, Invitrogen, Carlsbad, CA), and rabbit anti-Aβ 40-4 (1:2500, 1 h at RT, a kind gift of Dr. van Nostrand, Rhode Island University, Kingston, RI) followed by goat anti-rabbit IRDye800 (1:10000, 1 h at RT, Rockland, Pottstown, PA). Antibody solutions were prepared in Odyssey blocking buffer (LI-COR), diluted 1:1 in PBS. Between antibody incubations, membranes were washed extensively with PBST. Protein bands were visualized and band intensities were quantified using the Odyssey infrared imaging system (LI-COR).

### Thioflavin T Assay

Thioflavin T (ThT) was freshly dissolved in PBS before every experiment and filtered through a 0.22-μM filter. Aβ peptides were diluted to 10 μM in PBS containing 20 μM ThT and dispensed (100 μl) into a 96-well optical bottom black plate (VWR, Radnor, PA). Vehicle controls, containing 13 mM NaOH, were also diluted in PBS. To assess the effect of apoE on Aβ aggregation, apoE2, apoE3, or apoE4 were added to a final concentration of 0.1 μM. A Fluostar Optima plate reader (BMT Labtech, Ortenberg, Germany) with an excitation wavelength of 448 and emission wavelength of 482 was used to measure ThT fluorescence. The plate was incubated at 37 °C for 48 h and fluorescence was measured every 15 min, immediately preceded by 15 s of agitation. Fluorescence levels relative to ThT alone were calculated and normalized to the maximum fluorescence value.

### Cell Culture

Primary human cerebrovascular (leptomeningeal) smooth muscle cells (SMCs) and primary human cerebrovascular (leptomeningeal) brain pericytes (HBPs) were isolated from human brain tissue obtained at autopsy as described previously [31, 32] and maintained in EMEM supplemented with antibiotics, human serum (5% for SMCs; 10% for HBPs), 20% FCS, and 1 pg/ml human bFGF. Culture flasks were precoated with fibronectin. Primary human brain microvascular endothelial cells (hBMEC, ACBRI 376) were purchased from Cell Systems (Kirkland, WA) and cultured in EBM2 basal medium (Lonza, Basel, Switzerland) supplemented with FCS (5%), hydrocortisone (1.4 μM), ascorbic acid (5 μg/ml), chemically defined lipid concentrate (1%), human bFGF (1 ng/ml), HEPES buffer (10 μM), and antibiotics. Culture flasks were precoated with collagen I (150 μg/ml in PBS).

### MTT Assay

To assess the cytotoxic effects of Aβ on cerebrovascular cells, changes in metabolic activity were assessed with a MTT assay. Cells were cultured to confluence in 96-well plates coated with fibronectin (SMCs/HBPs) or collagen (HBMECs) and pre-incubated with EMEM (SMCs/HBPs) or EBM2 (hBMECs) supplemented with 0.1% BSA for a minimum of 4 h. Then, cells were incubated for 20 h with different Aβ peptides at a final concentration of 0.001–10 μM in EMEM-0.01%BSA or EBM2-0.01%BSA. Subsequently, Thiazolyl Blue Tetrazolium Blue (MTT, Sigma-Aldrich) was added at a final concentration of 0.8 mg/ml and cells were incubated for 3 h. MTT precipitates were dissolved in MTT solvent (isopropanol containing 0.1% NP-40 and 3 mM HCl) before absorbance was measured at 560 nm on a Tecan Infinity F50 plate reader. Experiments were performed in duplicate and results were expressed as percentage metabolic activity relative to untreated cells.

### Statistical Analysis

All statistical analyses were performed using Graphpad Prism 5 for windows (San Diego, Ca, USA), and IBM SPSS Statistics 25 (Armonk, NY, USA). ELISA and Western blot data for Aβ-apoE interaction were analyzed by two-way ANOVA, with apoE and Aβ isoforms as variables. The aggregation half times (t50), i.e., the times at which ThT fluorescence reached 50% of the maximum amplitude, were analyzed by ANOVA, followed by Bonferroni’s post hoc testing. MTT data were analyzed by ANOVA, followed by Dunnet’s post hoc testing.

## Results

### apoE-Aβ Complex Formation

**Fig. 1:**
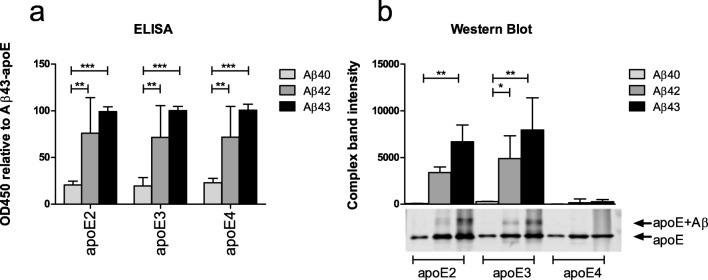
Aβ-apoE complex formation. The complex formation between different isoforms of Aβ and apoE was assessed semi-quantitatively using ELISA and SDS-PAGE/Western blotting. (**a**) Under the non-reducing conditions of the ELISA, apoE isoforms bound to the Aβ peptides in a comparable way. Aβ40 bound significantly less efficient to all apoE isoforms as compared with Aβ42 and Aβ43. (**b**) Under the reducing conditions of SDS-PAGE/Western blot analysis, Aβ40 bound less efficient to apoE2 and apoeE3 compared with Aβ42 and Aβ43. In addition, no interaction between apoE4 and any of the Aβ isoforms was observed using SDS-PAGE/Western blot analysis. The upper panel shows the quantification of the apoE-Aβ complex band of the blot that is shown in the lower panel. For ELISA experiments, samples were assessed in duplicates. Data represent mean (sd) of *n* = 4 (ELISA) and *n* = 2 (SDS-PAGE/Western blot) experiments. *p < 0.05; **p < 0.01; and ***p < 0.001 as analyzed by two-way ANOVA including apoE and Aβ isoform as variables

Semi-quantitative analysis of the degree of interaction between various apoE and Aβ isoforms under non-reducing conditions showed that the degree of interaction was only determined by the specific Aβ isoform, but not by the specific apoE isoform. Aβ43 did not differ from Aβ42 in its interaction with apoE, whereas Aβ40 had a significantly lower degree of interaction with apoE compared with Aβ42 (*p* < 0.01) and Aβ43 (*p* < 0.001, Fig. 1a).

SDS-PAGE/Western blot analysis provided information about the formation of Aβ-apoE complexes under more stringent conditions, that is, in the presence of SDS. Upon co-incubation of apoE2 or apoE3 with Aβ42 or Aβ43, an extra band at approximately 40 kDa was observed, detected both by antibodies directed against apoE and Aβ, indicating that a (SDS-resistant) protein complex of apoE and Aβ was formed. No complex formation between apoE4 and any of the Aβ isoforms was observed (Fig. 1b). The extra band was also not observed when analyzing samples containing only Aβ or apoE (data not shown). Comparing the intensities of these apoE-Aβ complex bands revealed a significantly weaker interaction between Aβ40 and apoE2 compared with Aβ43 and apoE2 (*p* < 0.01), and the interaction between Aβ40 and apoE3 was significantly weaker compared with both Aβ42 (*p* < 0.05) and Aβ43 (*p* < 0.01).

### Aβ43 Aggregation in the Absence and Presence of apoE

**Fig. 2:**
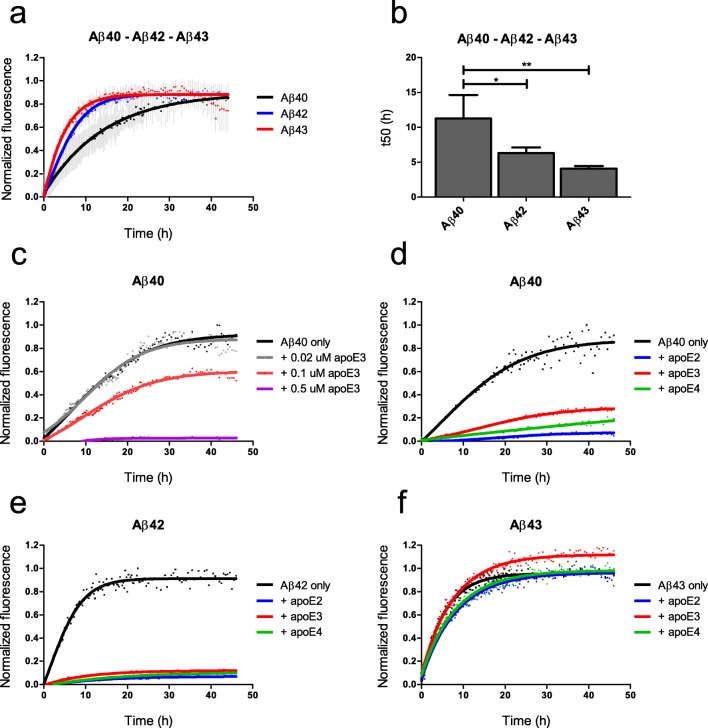
Aggregation kinetics of Aβ. Compared with Aβ42 and Aβ43, Aβ40 aggregated slower (**a**) and had a significantly higher t50 value, at which ThT fluorescence reached 50% of the maximum amplitude (**b**). ApoE3 inhibited Aβ40 aggregation in a concentration-dependent manner (**c**). Aggregation of Aβ40 (**d**) and Aβ42 (**e**) was inhibited by the addition of 0.1 μM apoE2, apoE3, or apoE4. Aβ43 aggregation was not affected by the addition of 0.1 μM of any apoE isoform (**f**). Aβ concentrations in all experiments were 10 μM. **a**, **b** Data represent mean (sd) of *n* = 4 experiments performed. **c**–**f** Representative data of *n* = 3 experiments. The t50 times were analyzed by ANOVA, followed by Bonferroni’s post hoc testing. *p < 0.05 and **p < 0.01

Normalized aggregation curves of Aβ42 and Aβ43 were comparable, whereas Aβ40 had a lower ThT incorporation rate (Fig. 2a). ThT fluorescence half times (t50), i.e., the time at which ThT fluorescence reaches 50% of the maximum amplitude, were significantly higher for Aβ40 (11.3 h) compared with Aβ42 (6.3 h, *p* < 0.05) and Aβ43 (4.1 h, *p* < 0.01; Fig. 2b). The addition of apoE3 to Aβ40 resulted in a concentration-dependent decrease of ThT incorporation (Fig. 2c). At a concentration of 0.1 μM, all apoE isoforms were comparably capable of inhibiting aggregation of Aβ40 (Fig. 2d) and Aβ42 (Fig. 2e). Aβ43 aggregation was not inhibited by any of the apoE isoforms (Fig. 2f).

### Effect of Aβ Isoforms on Cerebrovascular Metabolic Activity

**Fig. 3:**
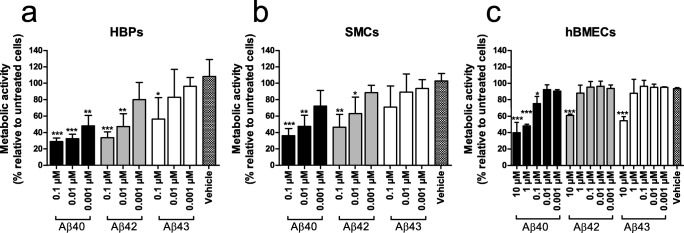
Effect of Aβ on metabolic activity of cerebrovascular cells. Aβ40, Aβ42, and Aβ43 reduced metabolic activity of HBPs (**a**), SMCs (**b**), and hBMECs (**c**) in a concentration-dependent manner. This effect was most pronounced for Aβ40, and least pronounced for Aβ43. HBMECs were less sensitive to Aβ compared with SMCs and HBPs, as only relatively high concentrations of 1 and 10 μM reduced metabolic activity. Data represent mean (sd) of 3 experiments. Data were analyzed by ANOVA, followed by Dunnet’s post hoc testing *p < 0.05; **p < 0.01; and ***p < 0.001 for Aβ-treated cells versus vehicle-treated cellsz

All Aβ variants had a concentration-dependent decreasing effect on metabolic activity of HBPs (Fig. 3a). However, the degree of this effect differed for the three Aβ isoforms; Aβ40 reduced metabolic activity at a concentration of 0.001 μM in HPBs (*p* < 0.01), whereas at this concentration, we observed no effect of either Aβ42 or Aβ43. Aβ42 reduced metabolic activity only at 0.01 μM (*p* < 0.01) or higher concentrations, whereas a concentration of 0.1 μM Aβ43 (or higher) was required to compromise metabolic activity of HBPs (*p* < 0.05). Direct comparison between the peptides revealed a significant difference only between 0.001 μM Aβ40 and 0.001 μM Aβ43 (*p* = 0.026).

Similar effects were observed in SMCs treated with Aβ peptides, although these cells were slightly more resistant to Aβ treatment (Fig. 3b). This may be due to biological variability (e.g., growth rate) that is inherent to the use of primary cells. At a concentration of 0.01 μM, metabolic activity of SMCs was reduced by Aβ40 (*p* < 0.01) and Aβ42 (*p* < 0.05). No effect of Aβ43 was observed at any of the tested concentrations. Direct comparison between the peptides revealed no significant differences between peptides.

Metabolic activity of hBMECs also decreased in a concentration-dependent manner as a result of Aβ treatment (Fig. 3c). However, these cells were less sensitive to Aβ treatment: Aβ concentrations needed to be 100-fold higher in order to induce a cytotoxic effect on hBMECs. A cytotoxic effect of Aβ40 treatment was observed at a concentration of 0.1 μM (*p* < 0.05) and higher (*p* < 0.001), whereas only at concentrations as high as 10 μM, metabolic activity of hBMECs was decreased by treatment of Aβ42 (*p* < 0.001) and Aβ43 (*p* < 0.001). A direct comparison between the peptides revealed a significant difference only between 0.1 μM Aβ40 and 0.001 μM Aβ43 (*p* = 0.044).

## Discussion

Some years ago, it has been shown that the Aβ43 peptide is highly abundant in the brains of AD patients and has neurotoxic properties [9, 10]. Interestingly, despite high levels in AD plaques, only very low Aβ43 levels in vascular deposits in CAA have been demonstrated [11, 12]. This observation may indicate that, compared with other Aβ species, the Aβ43 peptide has distinct properties, which prevent its accumulation in the cerebral vasculature, e.g., by more efficient clearance across the BBB. We assessed several characteristics of Aβ43 that may help to understand its preferred accumulation in plaques as opposed to cerebral vessels and related these to Aβ40 and Aβ42 characteristics. We analyzed Aβ43 in terms of its interaction with apoE, its aggregation propensity, and its toxicity towards cerebrovascular cells, including smooth muscle cells, pericytes, and endothelial cells.

A protein that is involved in the processes of Aβ aggregation, deposition, and clearance across the BBB is apoE []. However, despite many years of research and numerous studies, the precise role of apoE in the development of AD and CAA remains subject of investigation. Possession of the *APOE* ε4 allele is a strong risk factor for the development of both AD and CAA [33–35]. However, it is still not clear how apoE4 increases the risk of CAA and AD. An obvious explanation may be found in the interaction between apoE and Aβ, as these proteins are known to be able to form protein complexes [36]. In addition, while apoE2 or apoE3 appear to clear Aβ via the receptor LRP1, apoE4 seems to redirect Aβ clearance to the less efficient very-low-density lipoprotein receptor (VLDLR), possibly leading to slowing of this process [37]. These protein-receptor interactions have been suggested to contribute to the increased risk for developing AD and CAA seen in APOE ε4 carriers. Using SDS-PAGE/Western blotting, we observed that, unlike apoE2 or apoE3, apoE4 did not form complexes with Aβ, which is in line with previous observations [27, 38–41]. As SDS-PAGE/Western blotting is performed in the presence of SDS and therefore under relatively stringent conditions, this may indicate that the binding of apoE4 with Aβ is less stable compared with apoE2 and apoE3 and more easily disturbed by denaturing agents such as SDS [42]. In addition to the lower Aβ-binding properties of apoE4, we observed lower apoE-binding properties of Aβ40 compared with Aβ42 and Aβ43, both using ELISA analysis as well as the more stringent SDS-PAGE/Western blot analysis.

A particularly high aggregation speed might prevent Aβ43 from reaching the vasculature, due to immediate aggregation in the parenchyma. Alternatively, a low aggregation propensity might allow efficient clearance of monomeric Aβ43 across the BBB. The Aβ42 isoform is known to have increased hydrophobic properties and aggregation potential compared with Aβ40, due to the C-terminal addition of isoleucine and alanine [43, 44]. Our studies suggest that the addition of a neutral threonine does not affect the aggregation kinetics of Aβ43, compared with Aβ42, as has been reported before [11, 43, 45]. However, other studies have demonstrated an increased aggregation propensity of Aβ43 compared with Aβ42 [9, 10, 46]. Conversely, also decreased aggregation propensity of Aβ43 compared with Aβ42 has been reported [47]. These varying observations indicate that the aggregation properties of Aβ43 compared with the shorter Aβ peptides might not be straightforward and possibly could be dependent on the analytical method used and the source and concentration of Aβ. However, we are confident about the quality of our study since we carefully controlled for both reproducible conditions of the experiments and for the absolute amount of Aβ used in these aggregation studies, as we will discuss below.

Although many studies aimed to elucidate the effect of apoE on Aβ aggregation, the precise interaction between these proteins with respect to aggregation remains unclear. The effect of apoE may depend on the concentration of Aβ: at very high Aβ concentrations (∼ 80 to 300 μM), apoE has been reported to accelerate the fibrillization of Aβ [48–50]. However, at lower and more physiological Aβ levels (4–50 μM), apoE may have an inhibitory effect on Aβ aggregation [51–54], by inhibiting oligomerization [51, 52, 55] and, at higher concentrations, fibrillization of Aβ [51, 52]. Our findings of the inhibitory effect of apoE on relatively low concentrations (10 μM) of Aβ40 and Aβ42 aggregation support these observations. Only low, substoichiometric amounts of apoE (molar apoE:Aβ ratios of 1:100, absolute apoE concentrations of 100 nM) were required to block Aβ seeding or fibril growth. This suggests that apoE exerts its inhibitory effect not by binding to monomeric Aβ, but merely by blocking fibrillar Aβ growth sites [51, 52]. If apoE indeed has a higher affinity to bind Aβ fibrils as opposed to monomeric Aβ, this may explain the lower apoE-binding properties of (monomeric) Aβ40, compared with Aβ42 and Aβ43, which we observed with SDS-PAGE/Western blot and ELISA analysis. Since Aβ40 is less prone to oligomerization and aggregation than Aβ42 and Aβ43, it is likely more present in a monomeric state and therefore, it may bind less efficiently to apoE. Interestingly, in contrast to the effects of apoE on Aβ40 and Aβ42 aggregation, no inhibitory effect of apoE on Aβ43 aggregation was observed.

For our studies, we used the unlipidated recombinant (*E. coli*) form of human apoE, which may behave differently than lipidated apoE [56]. We repeated our experiments with apoE lipidated according to an established and published sodium cholate dialysis method [28]. Unfortunately, we found that the ThT aggregation assay is disturbed in the presence of lipid particles, hindering us to test the hypothesis that lipidation of apoE would result in an isoform-dependent effect on in vitro aggregation. We did assess the interaction between lipidated apoE and Aβ using ELISA, and SDS-PAGE and Western blotting, and found that lipidation of apoE did not affect the results (data not shown).

Increased toxicity of Aβ43, compared with Aβ40 and Aβ42, has been demonstrated in primary neurons and various cell lines, including SH-SY5Y cells, and PC12 cells [9, 10, 57, 58]. However, not all cells may be equally sensitive towards the effects of Aβ, which is illustrated by the recent observation that Aβ42 is much more toxic towards neurons compared with glial cells [59]. We studied, for the first time, the effects of Aβ43 on cerebrovascular cells, including SMCs, HBPs, and hBMECs. In SMCs and HBPs, we observed a toxic effect in the order Aβ40 > Aβ42 > Aβ43, which is in contrast to findings in neurons [10]. HBMECs were much less sensitive towards all Aβ isoforms; a toxic effect was only observed at high Aβ concentrations, again with a stronger effect for Aβ40 compared with Aβ42 and Aβ43. The lower vulnerability of endothelial cells to Aβ-mediated toxicity is not unexpected, as in CAA-affected cerebral vessels, the endothelial cell layer is usually well preserved [20, 60]. Interestingly, the degree of cytotoxicity exerted by the various Aβ isoforms towards cerebrovascular cells is consistent with the tendency of these peptides to accumulate in CAA (Aβ40 > Aβ42 > Aβ43 [61]). We speculate that lower sensitivity of cerebrovascular cells to Aβ43 may prevent its accumulation in cerebral vessel walls, although our data do not prove such a direct relation. Aβ-induced dysfunction and death of cerebrovascular cells (mainly SMCs) have been shown in several animal models of CAA [62–67]. The sequence of events during the development of CAA seems to entail initial deposition of Aβ in basement membranes in the tunica media of cerebrovascular arteries, followed by Aβ deposition at the cellular surface and replacement of the SMC layer and connective tissue [18]. Aβ deposition in arteries may lead to further CAA development through disruption of perivascular drainage [68], one of the clearance pathways of Aβ, in which SMCs seem to play a pivotal role [69]. An alternative explanation for lower Aβ43-mediated toxicity may be its fast aggregation, since larger Aβ aggregates have been shown to be less toxic towards cerebrovascular cells [70]. However, this is not a likely explanation since we did not observe different aggregation propensities for Aβ43 compared with Aβ42.

Receptor-mediated uptake of Aβ by cerebrovascular cells plays an important role in Aβ clearance, and differences in the uptake of the various Aβ peptides might also contribute to the low levels of Aβ43 in CAA. There are, however, indications that Aβ43, compared with Aβ40, is cleared less efficiently by cells of the *Drosophila* nervous system [71]. Furthermore, assessment of brain tissue of immunized AD cases may provide insight into clearance of Aβ peptides as immunotherapy leads to solubilization of Aβ parenchymal plaques but perseverance or even increase of CAA due to failing clearance mechanisms. However, assessment of Aβ levels in these cases did not reveal differences in cerebrovascular expression between Aβ43, and Aβ42 and Aβ40 [61]. From these studies, it may be speculated that low abundance of Aβ43 in CAA is not explained by more efficient clearance of Aβ43 across the BBB. However, mechanistic studies are needed to further elucidate the efficacy of active Aβ43 clearance from the cerebral vasculature, for example by assessing the binding affinity of Aβ43 for LRP1, and transport of Aβ43 across BBB-model systems.

A crucial requirement for the assessment of Aβ kinetics or cytotoxicity is the availability of a monomeric Aβ stock. The presence of pre-existing Aβ aggregates complicates the interpretation of data, and may also impede the reproducibility of findings. A common approach for the removal of pre-existing aggregates is treatment with hexafluoroisopropanol (HFIP) [72, 73], followed by resuspension in DMSO [74]. Despite this assumption, HFIP treatment has also been suggested to induce self-assembly of Aβ peptides [30, 75–77]. Another, frequently used, method to obtain monomeric Aβ solutions is pre-treatment with alkaline reagents such as NH_4_OH or NaOH, which prevents the Aβ solution of reaching the isoelectric point of 5.5 at which Aβ aggregation is maximal [78–80]. We followed a previously established protocol based on this latter method [30, 81–85] for the preparation of aggregate-free Aβ solutions and found that it tremendously increased reproducibility of our findings as compared with HFIP pre-treatment. Furthermore, we carefully controlled experimental conditions by determination and normalization of Aβ concentrations before every experiment, as we observed a high variation in actual protein content of commercially available Aβ peptides, often not consistent with the indicated amounts. We are confident that, by carefully controlling experimental Aβ input, we present highly reproducible and novel findings concerning several biochemical characteristics of Aβ43.

## Conclusions

We found that the extra amino acid residue(s) in Aβ43 alters the characteristics of this peptide compared with Aβ40 and Aβ42. We found that, despite strong interactions between Aβ43 and apoE as shown by ELISA and SDS-PAGE/Western blotting, at substoichiometric amounts, apoE does not inhibit Aβ43 aggregation. This is in contrast to our observation that the aggregation of both Aβ40 and Aβ42 was inhibited by the addition of apoE. As apoE is abundantly present in the brain, possibly, Aβ43 more readily aggregates and accumulates in the brain parenchyma leading to a reduced net transport towards the vasculature, which may explain its low levels in CAA. Furthermore, we demonstrated lower sensitivity of cerebrovascular cells towards Aβ43 compared with Aβ40 and Aβ42, which may also contribute to lower levels of Aβ43 accumulation in cerebral vessel walls. The results of this study suggest that differential aggregation propensity and cytotoxicity towards cerebrovascular cells may explain the relatively low abundance of Aβ43 in CAA.

## Abbreviations

Aβ: Amyloid-β
AD: Alzheimer’s disease
CAA: Cerebral amyloid angiopathy
APP: Amyloid precursor protein
SMCs: Smooth muscle cells
HBPs: Human brain pericytes
BBB: Blood-brain barrier
apoE: Apolipoprotein E
LRP1: Low-density lipoprotein receptor–related protein 1
RT: Room temperature
PBST: PBS containing 0.05% Tween20
OD: Optical density
ThT: Thioflavin T
EMEM: Eagle’s minimal essential medium
hBMEC: Human brain microvascular endothelial cells
MTT: Thiazolyl Blue Tetrazolium Blue
VLDLR: Very-low-density lipoprotein receptor
